# From Bench to Clinic: The 2024 FASEB Scientific Research Conference on NAD Metabolism and Signaling

**DOI:** 10.1186/s10020-025-01394-0

**Published:** 2025-10-31

**Authors:** Shin-ichiro Imai, Eija Pirinen, Michael N. Sack, Jonas T. Treebak, Charalampos Tzoulis, Santina Bruzzone, Andreas H. Guse, Michael O. Hottiger, Xiaolu A. Cambronne

**Affiliations:** 1 Washington University School of Medicine, St. Louis, MO USA; 2 Research Unit of Biomedicine and Internal Medicine, Faculty of Medicine, University of Oulu, Oulu, Finland; 3 Research Program for Clinical and Molecular Metabolism, Faculty of Medicine, University of Helsinki, Helsinki, Finland; 4 Medical Research Center Oulu, Oulu University Hospital and University of Oulu, Oulu, Finland; 5 Biocenter Oulu, University of Oulu, Oulu, Finland; 6 National Heart, Lung and Blood Institute, NHLBI, NIH, Bethesda, MD USA; 7 Novo Nordisk Foundation Center for Basic Metabolic Research, Faculty of Health and Medical Sciences, University of Copenhagen, Copenhagen, Denmark; 8 Department of Neurology, Neuro-SysMed, Haukeland University Hospital, Jonas Lies vei 65, Bergen, 5021 Norway; 9 Department of Clinical Medicine, University of Bergen, Pb 7804, Bergen, 5020 Norway; 10 K.G. Jebsen Center for Translational Research in Parkinson’s disease, University of Bergen, Pb 7804, Bergen, 5020 Norway; 11 University of Genova, Genova, Italy; 12 Department of Biochemistry and Molecular Cell Biology, University Medical Center Hamburg-Eppendorf, Hamburg, Germany; 13 Department of Molecular Mechanisms of Disease, University of Zurich, Zurich, Switzerland; 14 Department of Molecular Biosciences, The University of Texas at Austin, Austin, TX 78737 USA

## Abstract

The 2024 FASEB Scientific Research Conference on NAD Metabolism and Signaling was held in Lisbon, Portugal and served to (1) unite researchers, clinicians, and trainees, (2) create opportunities for early-stage investigators by showcasing their work on an international stage and promote collaborations, (3) train the next generation of scientists in the field, and (4) improve human health by furthering our understanding of NAD^+^ metabolism and signaling.

With the burgeoning potential of NAD^+^ as a therapeutic agent for multiple health conditions, as well as many remaining scientific questions about the NAD^+^ metabolome, an expert panel discussion titled “NAD^+^ Health Outcomes Forum: A Call to Action” was hosted on Thursday, August 29, 2024. The main objectives were to discuss and translate what is known about NAD^+^ biology into tangible actions and to identify what remains unknown into a research call to action.

Given the broad and reaching impact of NAD^+^ on health, there is significant interest in NAD^+^ pathway modulation, including through precursors such as nicotinic acid, nicotinamide (NAM), nicotinamide riboside (NR), and nicotinamide mononucleotide (NMN). There is also growing research regarding the heterogeneity among individuals, as well as differences and similarities among the NAD^+^ precursors, specifically in relation to dosing, timing, and their impact on various health conditions.

## Introduction

The biennial NAD^+^ Meeting Series started sixteen years ago and has successfully united the study of NAD^+^ biology across field-specific disciplines. The 2024 FASEB meeting was held August 25th − 29th in Lisbon, Portugal, and organized by Professors Andreas H. Guse (University Medical Centre Hamburg-Eppendorf, Germany), Santina Bruzzone (University of Genova, Italy), Michael O. Hottiger (University of Zurich, Switzerland), and Xiaolu A. Cambronne (University of Texas at Austin, USA). It hosted over 120 international researchers and clinicians with a shared interest in understanding NAD^+^-dependent mechanisms in metabolism and signaling, as well as evaluating the potential of this molecule for improving human health.

## Program elements

In 2024, the scientific program featured 31 invited speakers who anchored 9 scientific topic sessions (Table 1), plenary talks from Prof. Mathias Ziegler (University of Bergen, Norway) and Prof. Daniela Corda (National Research Council, Italy), 28 short talks selected from submitted abstracts, two poster sessions, and a moderated forum on clinical NAD^+^ research. The program included career-development elements such as trainee awards (Table 2), a meet-the-experts luncheon, and a facilitated discussion by Dr. Wendy Ingram from Dragonfly Mental Health titled “Mentoring as a Two-way Street”. We honored colleagues who had passed away since the last meeting: Professor Nathan A. Berger, Case Western Reserve University; Professor Timothy F. Walseth, University of Minnesota; and Professor Anthony A. Sauve, Weill Cornell Medical College.

**Table 1:** Scientific Sessions, Title and Order

1	NAD in cancer
2	Sirtuin mechanisms
3	NAADP Signaling
4	Biology of organellar NAD
5	NAD in aging
6	ADP-ribosylation
7	NAD in neurobiology
8	Clinical trials with NAD precursors
9	Late-breaking session

**Table 2:** Trainee Awardees of Top Poster and Short Talk Presentations

Benitez-Rosendo, Andres	Mayo Clinic Medical Center, Florida USA	Poster
Ferro, Valentina	Vrije University Amsterdam, Netherlands	Poster
Perez Mato, Raul	University of Genoa, Italy	Poster
Sandmann, Max	UMC Hamburg-Eppendorf, Germany	Poster
Sumi, Thoniparambil Sunil	Indian Institute of Science, Bangalore, India	Poster
Astigiano, Cecilia	University of Genoa, Italy	Short talk
Seifert, Sophie	German Cancer Research Center, Heidelberg, Germany	Short talk
Wu, Xiaoyue	National Institute of Environmental Health Sciences, USA	Short talk

Reflecting the global community of NAD^+^ researchers, participants traveled from over 20 countries across North and South America, Europe, Asia, and Australia. US attendees arrived from 21 distinct states (53% of attendees from non-coastal states), and European attendees arrived from 11 distinct countries. The meeting encompassed the expanding NAD^+^ metabolome, including NAD^+^, NADH, NADP^+^, NADPH, NAADP, ADP-Ribose (ADPR), NAM, methylated-NAM, and pyridone nucleotides. Approaches scaled from atomic structural analyses to human physiology, and represented fields included chemistry, biochemistry, molecular and cell biology, computational biology, human and organismal physiology, synthetic biology, and engineering.

## Emerging themes

The scientific meeting encompassed established topics in NAD Metabolism and Signaling, such as intracellular NAADP-mediated Ca^2+^ signaling, DNA damage and repair, aging, and epigenetic mechanisms. In 2024, we featured new scientific sessions focused on human research, cancer, and neurobiology. In addition, several new NAD-related molecules were reported at this meeting, including new variants of cyclic ADPR (cADPR), ribosylated pyridones, trigonelline, and new roles for O-acetyl-ADPR. Compartmentalization of these signals and pathways was a common thread throughout the meeting. A major benefit of the meeting was the integration of ideas and collaborative outcomes across disciplines, highlighted by the recurrence of concepts across scientific sessions. Emerging themes are discussed below.

### NAD^+^ and drug-resistant cancers

One theme that emerged was the concept of targeting NAD^+^ biosynthesis and consumption to combat drug-resistant cancers. Session talks from Santina Bruzzone, Valentina Audrito (University del Piemonte Orientale, Italy), Sophie Seifert (German Cancer Research Center, Germany), and Xiaoyue Wu (National Institute of Environmental Health Sciences, USA) focused on biosynthetic pathways as vulnerabilities. Presentations from Sridevi Challa (University of Chicago, USA), Alejandro Vaquero (Josep Carreras Leukaemia Research Institute, Spain), Giovanna Grimaldi (National Research Council Naples, Italy), Caterina Miro (University of Naples Federico II, Italy), and Michael O. Hottiger highlighted new roles of NAD^+^-consuming enzymes in tumorigenesis.

### Intracellular regulation

Mechanisms for how the intracellular NAD^+^ metabolism is regulated, compartmentalized, and function with other metabolic pathways were highlighted by presentations from Xiaolu Cambronne (University of Texas at Austin, TX), Gerta Hoxhaj (UT Southwestern Medical Center, TX), Denis Titov (University of California Berkeley, CA), Lena Høyland (University of Bergen, Norway), Tanner Wright (Ludwig Maximilian University, Germany), Michael Cohen (Oregon Health & Sciences University, OR), and Lee Kraus (UT Southwestern Medical Center, TX). Included were presentations from the forefront about NAD^+^ in bacterial immunity by Ilya Osterman (Weizmann Institute of Science, Israel) and in RNA modification by Karla L. Feijs-Zaja (RWTH Aachen, Germany).

### Cardiovascular health

NAD^+^ consumption by Sirtuins, ADP-ribosylases, and Sterile alpha and TIR motif containing 1 (SARM1) in cardiovascular biology was covered in talks by Nagalingam Ravi Sundaresan (Indian Institute of Science, Bangalore, India), Arushi Taneja (Indian Institute of Science, Bangalore, India), and Chi Fung Lee (Oklahoma Medical Research Foundation, OK), respectively. Roger Ottenheijm (University of Heidelberg, Germany) presented a new model for NAADP-mediated calcium release. Contributions of NAD^+^ biosynthesis and regulation in the heart were reported in talks by Ann Chiao (Oklahoma Medical Research Foundation, OK) and Joseph A. Baur (University of Pennsylvania, PA).

### Neurodegeneration

Mechanistic roles for NAD^+^-consuming enzymes SIRT6 and SARM1 in neurodegeneration models were presented by Deborah Toiber (Ben-Gurion University of the Negev, Israel), Yong Juan Zhao (The Chinese University of Hong Kong, Shenzhen, China), and Michael Coleman (University of Cambridge, UK). Cecilia Astigiano (University of Genova, Italy) discussed NAD^+^ pathway components as biomarkers in a swine model of Amyotrophic Lateral Sclerosis. Vilhelm Bohr (University of Copenhagen) and Charalampos Tzoulis (University of Bergen, Norway) discussed preliminary results from clinical trials for Werner syndrome, ataxia, and Parkinson’s Disease.

### Inflammation, infection, and immunity

Multiple exciting presentations touched on NAD^+^-driven signaling and states in models of inflammation, immune cells, and infection. New mechanistic insights were presented about NAADP signaling from Andreas Guse, Jonathan Marchant (Medical College of Wisconsin, WI), and Susanna Zierler (Johannes Kepler University Linz, Austria). Roles in innate immunity for consuming enzymes SARM1, CD38, and ADP-ribosylases were presented by Ryoichi Sugisawa (Kindai University, Japan) and Claudia Chini (Mayo Clinic College of Medicine, FL). Julianna Dias Zeidler (Universidade Federal do Rio de Janeiro, Brazil) discussed NAD^+^ in Zika virus-induced neuroinflammation, and Ralf Fliegert (University Medical Center Hamburg-Eppendorf, Germany) discussed attenuating SARS-CoV-2 infection. NAD^+^-driven immunometabolism and inflammation were described by Michael Sack (National Heart, Lung, and Blood Institute, USA) and for treating inflammatory bowel disease by Wheeseong Lee (Lmito Therapeutics Inc., Korea).

### Physiology and aging

With the spurred interest in age-dependent changes in NAD^+^ metabolism, there were multiple talks on aging. These presentations included novel concepts and new research models and was anchored by talks from Melanie McReynolds (Pennsylvania State University, PA), Shin-ichiro Imai (Washington University School of Medicine in St. Louis, MO), Eduardo Chini (Mayo Clinic College of Medicine, FL), Ralph Meyer (Utah State University, UT), Takashi Nakagawa (University of Toyama, Japan), and Xiaoling Li (National Institutes. Data regarding new precursors were presented by Yue Yang (Weill Cornell Medicine, NY) and Vincenzo Sorrentino (National University of Singapore, Singapore).

Discovered roles for NAD^+^ in a range of human physiology from pregnancy to liver regeneration to addiction were represented by talks from Charles Brenner (Beckman Research Institute of the City of Hope, CA), Sarmistha Mukherjee (University of Pennsylvania, PA), Andrea Benzi (University of Genoa, Italy), Eija Pirinen (University of Oulu, Finland), Stephen Gardell (Advent Health Translational Research Institute, FL), Jonas T. Treebak (University of Copenhagen, Denmark), and Hartmut Cuny (Victor Chang Cardiac Research Institute, Australia). This was complemented by new developments in methodology presented by Liliya Euro (NADMed, Finland), Helen Dauben (Max Planck Institute for Biology of Ageing, Germany), Yasmeen Nkrumah-Elie (ChromaDex, USA), and Marie Migaud (University of South Alabama, AL), whose approaches identified new functional molecules from NAD catabolism.

## NAD^+^ health outcomes forum

The timeliness of the meeting was well-positioned to capture the excitement surrounding the inaugural studies in humans about the feasibility, usefulness, and safety of NAD^+^ modulation to counter disease. A special panel, NAD^+^ Health Outcomes Forum: A Call to Action, was arranged to promote discourse on the burgeoning potential of NAD^+^modulation as a therapeutic option for a myriad of health conditions as well as many remaining questions around clinical applications for NAD (Zapata-Pérez et al. 2021; Migaud et al. 2024; Chini et al. 2021). Dr. David Katz moderated the session. The panelists were Drs. Eija Pirinen, PhD, Shin-ichiro Imai, MD, PhD, Michael Sack, MD, PhD, Jonas T. Treebak, PhD, and Charalampos Tzoulis, MD, PhD. Included here is a special summary report of the discussion among panelists. We summarize key consensuses and variances in Table 3.

The panelists agreed the following topics are the most pressing issues to address withrespect to clinical applications for NAD biology.

**Table 3:** Summary of NAD+ health outcomes forum: a call to action

Topic﻿	Recommendations
NAD^+^ biomarkers andreal-time NAD^+^ measurement assays﻿	§ Panelists described that improved biomarkers and assays would better determine NAD^+^ pathway activation and levels. § Levels may vary by organ/tissue, and site-specific assays will be of value.
NAD^+^ precursors﻿	§ Panelists agreed that additional research is needed to determine which NAD^+^ precursor is best for various conditions and diseases aswell as for understanding tissue-specific effects. § Many panelists concurred that the lack of head-to-head studies comparing the effects of the different NAD^+^precursors make it challenging to determine which NAD^+^ precursor to recommend.
NAD^+^ route of administration	§ Panelists stated that testing routes of administration is extremely important to better understand NAD^+^pathway activation.
Clarification of NAD^+^ recommendations	§ Panelists concurred that more evidence is needed from phase three or four clinical trials before NAD^+^therapy recommendations are made for the general public. To complement this work, some panelists suggested conducting retrospective, observational studies involving individuals taking NAD^+^ precursors to examine the impact of NAD^+^ on the general population. § There would be value in establishing efficient, reliable ways to correlate dosing of any given NAD^+^precursor with tissue levels § Several panelists affirmed that NAD^+^ therapy would be beneficial in rare and mitochondrial diseases as well as for conditions that have early clinical evidence (i.e. ataxia telangiectasia) and those for which there is plausible link to the NAD^+^ pathway and want of established treatments.
Propose an NAD^+^ taskforce among researchers running clinical trials for:﻿	§ Increased cohort sizes§ Pooled resources§ Alignment of sample collection and analyses § Examination of different disease states§ Expedited comparisons of NAD^+^ precursors

### Unknowns needing further exploration

Discussion began around the main unknowns surrounding NAD^+^ biology. Charalampos Tzoulis highlighted the interindividual differences and the heterogeneity across individuals in the NAD^+^ response as being two important unknowns. In addition, he speculated that mapping specific factors that demonstrate interindividual differences in response to NAD^+^ boosting could help personalize NAD^+^ treatment in the future.

Shin-ichiro Imai discussed a variety of unknowns in the field including the various pharmacokinetic effects of NAD^+^ boosting, the transport mechanisms of the different NAD^+^ precursors, the impact of NAD^+^ on the microbiome, the effect of a pre-existing NAD^+^ deficiency on conditions like Parkinson’s and Alzheimer’s disease, NAD^+^ responders versus NAD^+^ non-responders and the effects of NAD^+^ precursors on healthy individuals.

Eija Pirinen also underscored the need for more research around the mechanisms behind a pre-existing NAD^+^ deficiency. Pirinen stated that more evidence is essential to determine which NAD^+^ precursors are more effective among individuals and why. She further discussed the best approaches to correct an NAD^+^ deficiency, noting that mechanistic information behind a pre-existing NAD^+^ deficiency can be used to develop more targeted treatments.

Michael Sack concurred with Imai regarding the need for a greater understanding around NAD^+^ responders versus NAD^+^ non-responders. He also underscored the need for more research on the role of different organ systems in NAD^+^ responses. Lastly, Jonas Treebak posited that better measurements are needed to dynamically predict NAD^+^ responsiveness.

### No “one-size fits all” for the general population

The conversation shifted to discussing boosting NAD^+^ for the public through various approaches, focusing on the ingestion or infusion of NAD^+^ precursors. However, Treebak found it difficult to justify a general population NAD^+^ recommendation. For example, Treebak cited an association study in over 1300 individuals, which found that the participants with the highest NAD^+^ blood levels had the most features of the metabolic syndrome. (Liu et al. 2023) Treebak further postulated that if an individual does not have an obvious NAD^+^ decrease, it is hard to justify NAD^+^ supplementation.

Imai underscored that since interest in NAD^+^ has increased, more individuals are taking NAD^+^ boosting supplements (Kitzmiller 2024; Hamlette 2024). Because of this, he stressed the need for both retrospective and prospective studies involving individuals taking NAD^+^ precursors to examine the impact of NAD^+^ supplementation on general health. Tzoulis agreed with the need for those studies and emphasized the utility of pivotal phase 3 clinical trials.

### Multiple approaches for harnessing the NAD^+^ pathway

The dialogue moved to strategies that influence the NAD^+^ pathway. Pirinen kicked off the discussion, focusing on the relationships between NAD^+^ precursors and NAD^+^ levels as well as their downstream effects, including improved mitochondrial activity and suppressed inflammation.

Sack emphasized the use of different “-omic” approaches in big data to help understand which NAD^+^ pathways are involved. He also highlighted additional challenges surrounding the NAD^+^ pathway in different organ systems or diseases. Given these challenges, Sack underscored the need for an unbiased “-omic” approach to determine which specific features of NAD^+^ biology are playing a role in particular organs or diseases.

Treebak recommended focusing on identifying diseases where NAD^+^ levels are decreased in specific cells or tissues; for instance, skeletal muscle NAD^+^ levels in diseases such as sarcopenia and mitochondrial myopathy have been shown to be reduced (Migliavacca et al. 2019). He posited that this approach could identify beneficial scenarios for NAD^+^ precursors to boost NAD^+^ levels. Additionally, he cited a potential target ARTC1, an arginine-specific ecto-ADP-ribosyltransferase, which is involved in regulating muscle structure and sarcolemma integrity, and its activity is NAD^+^-dependent ((Leutert et al. 2018)–(Zhao et al. 2005)). Measuring downstream markers of ARTC1 activity could indicate if providing NAD^+^ precursors would result in positive muscle outcomes.

### Therapeutic NAD ^+^amounts depend on context

Katz moved the conversation to focus on defining therapeutic NAD^+^ levels. Treebak opened the discussion, giving a daily recommended dose of oral vitamin B3 as 14 milligrams for women and 16 milligrams for men, which is based on how NAD^+^ metabolites are secreted in the urine each day (Freese 2023). Tzoulis further expanded upon this and recommended using well-established vitamin B3 intake levels that have been shown to prevent deficiencies like pellagra (Dietary 2024).

Additionally, Tzoulis outlined another potential option by posing the question: Could certain age-related diseases be prevented by increasing NAD^+^ precursor intake? He further detailed that research first needs to establish whether increasing NAD^+^ levels has a health benefit. Once this evidence is shown, Tzoulis stated that target NAD^+^ levels could be established. He cautioned that these levels may not be the same for different populations and stressed the need to create studies based on larger data sets, which could be done by pooling resources. Tzoulis concluded that the current research does not have enough evidence to make an optimal NAD^+^ precursor dose recommendation, but he is hopeful that in 5–6 years, another panel could be convened to make such recommendations.

Rounding out this discussion, Katz highlighted the distinct differences between recommendations to prevent deficiencies versus recommendations to support optimal health. He cited the US Dietary Reference Intakes (DRIs) as an example (Dietary 2024), which are reference values for nutrients established to prevent deficiencies versus sustaining optimal health.

Pirinen agreed with Tzoulis regarding the utility of establishing reference values and expanded the need to have blood and tissue-specific target NAD^+^ levels. Moreover, she postulated that these levels could help develop treatment options to identify disturbed NAD^+^ homeostasis. However, she outlined some potential challenges such as measuring NAD^+^ levels reliably as well as genetic and gender factors that impact NAD^+^ levels.

Sack added to the discussion by citing that circadian rhythms affect NAD^+^ levels (Ramsey et al. 2009; Nakahata et al. 2009; Peek et al. 2013; Levine et al. 2022). Additionally, he questioned how diet and environment impact baseline and optimal NAD^+^ levels, which are not defined. Katz followed up on this topic by suggesting that optimal NAD^+^ levels could be explored in individuals experiencing optimal health spans, such as healthy centenarians living in Blue Zones (Candal-Pedreira 2025). He detailed that these populations have been extensively profiled by ethnographers. Katz described that the utility of testing in Blue Zones would not be limited to testing people who are over 100 years of age, but also to people from healthy, long-lived populations where 100 is routinely reached, with testing at varying ages to help determine age-specific “optimums”. However, Katz was unsure if this population had been used to ascertain the ideal target levels of different biochemical pathways for optimal health span.

Imai responded to this line of discussion, stating that determining optimal target amounts of NAD^+^ precursors will require a careful assessment of concurrent molecular biomarkers. As each individual has a variable baseline level and distinct metabolic demand for NAD^+^, an absolute measurement of the net metabolite level after administration may not accurately reflect health or the effects of supplementation. He concluded that there is a need for molecular biomarkers to assess what would constitute therapeutic NAD^+^ levels.

### More research needed on precursors and the NAD ^+^ pathway

The discussion moved to NAD^+^ precursors. Treebak kicked off the conversation citing the lack of head-to-head studies comparing the different precursors. Additionally, he referenced two recent studies to further elaborate on the challenges of comparing NAD^+^precursors. One study evaluated the impact of NMN in postmenopausal women and determined that NMN increased insulin sensitivity (Yoshino et al. 2021; Brenner 2021; Klein and Yoshino 2021). The other trial performed in men examined the effects of NR and found no impact on insulin sensitivity (Dollerup et al. 2018). According to Treebak, these studies highlight that trial-to-trial variability, sex differences and precursor differences all play roles in influencing the NAD^+^ pathway.

Continuing, Pirinen affirmed that all NAD^+^ precursors (nicotinic acid, NAM, NR, NMN) have been shown to increase NAD^+^ levels in at least one context. As such, she recommends studying all the precursors. Pirinen also acknowledged that the best approach would be to develop a clinical trial that tests all the precursors in a head-to-head manner. This research could reveal whether one precursor is more effective for a specific disorder or condition, while another precursor is more effective for a different disease or condition.

Imai followed, stating that nicotinamide increases bioenergetic activity everywhere, while NR and NMN have tissue-specific effects. Further, he cited research that showed that NR and NMN are transported through distinct carriers (Grozio et al. 2019; Kropotov et al. 2021; Ratajczak et al. 2016). He speculated that the tissue distribution of transporters could help define which precursor to use. Additionally, he ventured that when NMN or NR pass through transporters, there may be other cell signaling pathway effects that are not fully understood at this moment.

Tzoulis rounded out this topic of discussion by agreeing with the other panelists that the only way to compare NAD^+^ precursors is through head-to-head clinical trials. He conceded that this approach would be extremely challenging, given the need for large sample sizes (potentially more than 1,000 participants per arm) because the study would not be testing against a placebo and the differences in effect would be small. Tzoulis concluded that there are currently many different clinical trials in progress looking at different disease conditions and different NAD^+^ precursors. He recommended letting the data emerge from the trials, and when there is compelling phase three clinical evidence in one or several diseases, additional research can be completed to tease out which NAD^+^ precursor is the best. Furthermore, he concluded that the other precursors could be tested with a non-inferiority trial (Definition 2024).

### Route of administration for NAD ^+^ and its precursors

The conversation shifted to the route of administration, as intravenous (IV), subcutaneous, intramuscular (IM), oral, nasal and transdermal are all possible options for delivering NAD^+^ and its precursors. Treebak opened the discussion citing a study comparing NR delivery (oral versus IV) in mice (Damgaard et al. 2022). When NR was delivered orally, NAD^+^ increased in the liver but not in the muscle or adipose tissue. However, when NR was given via IV, NAD^+^ was elevated in the muscle and adipose tissue, but not in the liver (Damgaard et al. 2022). Further, he cited that the route of administration can target specific cell types. Treebak also discussed the importance of having routes of administration that bypass the microbiome (Shats et al. 2020; Chellappa et al. 2022; Yaku et al. 2025). He suggested using metabolite carriers (i.e., use of M cells in the intestine to enter the lymphatic system) as a potential strategy.

Concurring with Treebak, Tzoulis emphasized that testing routes of administration is extremely important. He also added that if bypassing the microbiome is a good strategy, IV delivery would be the best approach. However, Tzoulis questioned the need for IV NAD^+^ therapy as this would only boost NAD^+^ levels acutely, which given the panel’s discussion around the use of NAD^+^ in chronic diseases, would have less clinical impact.

Pirinen centered her discussion on the best administration route by citing their recent clinical trial with NR (Lapatto et al. 2023). In this study, BMI-discordant twins supplemented with NR showed an increase in muscle NAD^+^ biosynthesis, with a weaker effect on white adipose tissue. Further, the study did not demonstrate any mitochondrial changes in white adipose tissue but did detect mitochondrial changes in the muscle. Pirinen suggested that oral administration of NR may not be the best method of delivery when targeting white adipose tissue.

The conversation with Imai shifted to potential methodological issues. He flagged that important compounds such as ADP-ribose, cyclic ADP-riboses (cADPRs), and NAADP, were missing from the methodological development discussion. These molecules are key second messengers involved in various cellular signaling pathways (Fliegert et al. 2007). Imai expressed concern for the lack of measurement because as NMN infusion is rising in popularity, this likely increases the generation of these second messengers. In particular, NMN can be a strong activator for SARM1, an enzyme that plays a key role in neurodegenerative disorders and inflammation (Fliegert et al. 2007; Figley et al. 2021; Loreto et al. 2015; Zhao et al. 2019). SARM1 activity is sufficient to produce these second messenger products in vitro (Essuman et al. 2017; Shi et al. 2022; Bratkowski et al. 2020; Osterloh et al. 2012; Jiang et al. 2020; Gerdts et al. 2015). He continued that in the context of an NMN or an NR infusion, NR is not known to activate SARM1, whereas NMN does (Fliegert et al. 2007; Figley et al. 2021; Loreto et al. 2015; Zhao et al. 2019). According to Imai, this suggests that an NR infusion may be safer, if it is not converted to NMN. However, he cautioned that if second messenger levels increase after an infusion of NR or NMN, he would oppose the infusion of all these precursors. Since there is currently no clinical methodological assay developed for ADPR and cADPR levels, he concluded that it is not possible to definitively select a safe NAD^+^ precursor for infusion.

Tzoulis followed up on this topic, highlighting that more precise methods are needed to reliably measure NAD^+^ levels in the human brain in vivo. Current protocols use phosphorus magnetic resonance spectroscopy (^31^P-MRS), but this technique has limitations, including partially overlapping NAD^+^ and NADH spectra, as well as low signals without specialized instrumentation (Brakedal et al. 2022; Payne et al. 2023; Zhu et al. 2015). Moreover, he detailed that while cerebrospinal fluid (CSF) has been used to measure NAD^+^ levels, NAD^+^ levels are usually very low in the CSF. He added that while downstream CSF metabolites could be used, such as 2PY or 4PY, these markers do not provide the most accurate measurement because they could be derived from NAD^+^ in the brain or the metabolites could have entered from blood leaking into the CSF. Additionally, routine testing through CSF collection has significant limitations due to an invasive collection method. Given this, Tzoulis reiterated the need for precise, reliable and less invasive ways to quantify real-time, in vivo NAD^+^ levels in the human nervous system.

This discussion was rounded out by Treebak, who discussed proton magnetic resonance spectroscopy (^1^H-MRS). Since ^1^H-MRS has been used to detect and quantify NAD^+^in the brain, he posited that this technique could be applied to other tissues (Dziadosz et al. 2022). Treebak concluded that in general, there is a significant need for better in vivo dynamic measurements of NAD^+^ since current measurements are from isolated tissue collection.

### Are there conditions amenable to NAD^+^ therapy?

Katz shifted the panel to focus on disorders and conditions that would have the most therapeutic benefit from NAD^+^ boosting. Sack kicked off the conversation by focusing on cardiology, autoimmunity, and inflammation. He concluded that it may be difficult to demonstrate a benefit for these conditions currently that are above and beyond the standard of pharmacotherapy care.

Imai stated that he does not recommend developing NMN as a pharmaceutical drug because prescription by physicians would be necessary to use it. Instead, he proposed that NAD^+^ precursors could be used with correct guidelines in aging populations to prevent age-associated dysfunctions and diseases before they develop. Further, he posited that this could result in lower costs, fewer side effects and less need for multiple pharmaceutical drugs. Given this potential, he stressed the need for evidence-based nutraceutical health promotion research.

Pirinen stated that NAD^+^ boosting therapy could be efficacious in rare diseases that lack curative treatments. She cited mitochondrial disorders as an example, referencing previous research showing the impact of nicotinic acid on mitochondrial myopathy (Pirinen et al. 2020). Pirinen agreed with Imai that the use of NAD^+^ boosting therapy as a nutraceutical for preventing diseases and conditions, particularly obesity, would be a compelling area of study.

However, Katz expressed caution around this point. He stated that in conditions where highly effective pharmacotherapies are available the demonstration of an additional health benefit would be very challenging. Yet, for at-risk populations without highly developed pharmacotherapy plans, they both agreed that optimizing metabolic pathways via NAD^+^ boosting therapy could be beneficial.

Tzoulis stressed that no recommendations on NAD^+^-based therapy can be made before systematic and adequate clinical evidence from regulatorily approved trials has been presented. He further stated that the only medical condition where we currently have irrefutable evidence of efficacy for NAD^+^-therapy is pellagra, a condition caused by NAD^+^ deficiency. In addition, he agreed with Pirinen, affirming that NAD^+^ boosting therapy may be beneficial in rare diseases, mitochondrial diseases, and diseases such as ataxia telangiectasia for which some clinical evidence has been reported (Presterud et al. 2024) while larger studies are awaited. He further detailed that in cases of rare diseases that prohibit large clinical trials, and where robust Phase 2 efficacy evidence is available, recommending NAD^+^ therapy may be considered under appropriate clinical supervision. Otherwise, for other conditions and populations, Tzoulis suggested waiting for Phase 3 clinical evidence. He ended his discussion by focusing on the preventative potential of NAD^+^. He agreed that there is significant potential, but again, stressed the need for rigorous clinical research and evidence. Further, Tzoulis suggested that examining the preventive potential of NAD^+^ supplementation in individuals who are at a higher risk of neurodegenerative diseases is important.

Treebak agreed with the list of conditions already discussed and added that energetic stress (i.e., reductions in cellular ATP levels) can be induced by excessive amounts of NAD^+^ precursors. Furthermore, he stated that while exercise is the best way to induce beneficial adaptations across all cells in our body, a combinatory approach could be used with NAD^+^ precursors and exercise. In addition, Treebak speculated that these effects could be additive. He also emphasized that this outcome could be particularly therapeutic in the context of sarcopenia, an age-associated condition characterized by loss of skeletal muscle mass and strength. As such, Treebak concluded that NAD^+^ boosting therapy could improve mobility and muscle function, which could motivate individuals to be more active, resulting in a cumulative result.

Continuing this topic, Tzoulis agreed that the research surrounding the additive effects of NAD^+^ precursor administration with exercise is underexplored. Further, he added that his research group is planning to start a preventive trial using NAD^+^ replenishment for individuals in the prodromal phase of Parkinson’s disease and related disorders, for example, identified by the presence of isolated rapid eye movement (REM) sleep behavior disorder (iRBD) – a condition strongly associated with future development of these diseases (Postuma et al. 2019). He explained that his group will also examine the impact of NAD^+^ replenishment alone, or in combination with exercise. Tzoulis described that the study will encourage exercise through gamification. This will be done by partnering with health technology companies that encourage participants to exercise while measuring actual exercise levels.

Exercise was further discussed by Imai, who agreed that this area of research is particularly compelling. He detailed recent research that showed that moderate exercise significantly increased circulating extracellular nicotinamide phosphoribosyltransferase (eNAMPT) levels in humans, a rate-limiting enzyme in the NAD^+^ biosynthetic pathway (Chong et al. 2022). Imai added that his group is currently exploring the impact of NAD^+^ precursors on circulating levels of eNAMPT.

### Translating the NAD^+^ science into recommendations

To conclude the panel discussion, Katz asked the group to discuss what should be done with the current understanding of NAD^+^ metabolism. Sack started the discussion by stating that the decision to use NAD^+^ therapy depends on the patient population and their economic circumstances. For example, if patients have limited economic resources, payments for guideline-directed therapy should take precedent over vitamin supplementation.

When asked about a general public recommendation for NAD^+^ precursors and their dosage or timing, Imai said that he usually gives lengthy and often intentionally blurred suggestions. However, he conceded that there was compelling evidence to take NAD^+^ precursors in the morning, given the circadian oscillation of NAD^+^ in humans (Ramsey et al. 2009; Nakahata et al. 2009; Peek et al. 2013; Levine et al. 2022). Imai ended his discussion stating that he is strongly opposed to NMN infusions due to the risk of activating SARM1 as previously discussed.

In the context of translating the NAD^+^ science into recommendations, Pirinen again stressed the need to wait for data from phase three or four clinical trials. She reiterated that the strongest recommendations surrounding NAD^+^ boosting therapy are for individuals with mitochondrial disorders and rare diseases. Pirinen further elaborated that if a patient has a mitochondrial disorder, she advises clinicians to explore whether the patient could have an NAD^+^ deficiency, whether NAD^+^ levels could be tested somewhere, and whether NAD^+^ precursor supplementation would be beneficial. Pirinen remained optimistic that perhaps at the next FASEB meeting, stronger recommendations could be made for clinicians.

For the general population, she stressed the importance of waiting until research shows how NAD^+^ precursors are affecting different health populations before developing recommendations and messaging. Additionally, for overall health, Pirinen advised waiting to take NAD^+^ precursors and instead focusing on modifying diet and physical activity. She concluded that it is still beneficial to follow developments and advancements in the field of NAD^+^ biology.

Tzoulis agreed with Pirinen regarding the need to wait for more data from large Phase 3 or 4 clinical trials (Zhang et al. 2025). Further, he stated that patients should refrain from using doses of NAD^+^ precursors that exceed approved daily recommendations for each product, as safety for higher doses has not yet been estabished. He added that patients with a chronic condition and potentially using other medications, should always discuss the use of NAD^+^ precursors or other supplements with their treating physician first.

Treebak rounded out the discussion acknowledging that the public encounters news around the effects of NAD^+^ and NAD^+^ precursors on various health conditions. As such, he recognized that the public may not be as patient as researchers and thus recommended that individuals try NAD^+^ boosting therapy to see if it works for them. He envisioned that some individuals would respond to NAD^+^ precursor treatment and based on the lack of evidence of detrimental effects of high doses of NR and NMN in clinical studies, it would likely be safe to consume 250 milligrams of these precursors each day (Dollerup et al. 2018; Berven et al. 2023; Yi et al. 2023).

With these individual conclusive remarks, the panel discussion was successfully concluded and was opened to the audience for a question-and-answer session.

## Conclusions

The 2024 FASEB SRC on NAD^+^ Metabolism and Signaling overcame barriers, united sub-disciplines, and promoted new collaborations. The meeting attracted attendees from various fields for a discourse and exchange of innovative ideas.

86% of attendees worked in academia, and 14% of attendees worked in industry. Within the academic sector, attendees represented large research universities, small colleges, research institutes, government labs, and hospital systems; 17% worked in smaller academic institutions with fewer than 5000 students. Three scientific talks were given by researchers employed by industry.

Among the speakers (26 from North and South America, 26 from Europe, 8 from Asia, and 1 from Australia), 49% (30/61) were female, 56% (34/61) were at an early career stage, and 61% (37/61) were new invitees defined by not having presented in the last 4 years. We note lower participation rates from specific geographical regions, such as Australia and none from Africa, as an area of improvement. Post-meeting attendee feedback indicated a 94% satisfaction rate, with enthusiasm for future meetings and recommendations to colleagues regarding the scientific content.

The 2024 FASEB SRC on NAD^+^ Metabolism and Signaling expanded its strong foundation in molecular mechanism to include robust discussions about clinical applications. In short, this 2024 meeting has accelerated the pace of discovery, expanded NAD^+^ research into new areas, and resulted in forward progress for the field.

The next 2026 FASEB NAD^+^ Metabolism and Signaling Scientific Research Conference is planned for June 7-11th, 2026 in Melbourne, Florida USA.

## Data Availability

No datasets were generated or analysed during the current study.

## Abbreviations

NAD^+^: Nicotinamide adenine dinucleotide
NADH: Reduced Nicotinamide adenine dinucleotide
NADP+: Nicotinamide adenine dinucleotide phosphate
NADPH: Reduced Nicotinamide adenine dinucleotide phosphate
NAADP: Nicotinic acid adenine dinucleotide phosphate
ADPR: Adenosine diphosphate ribose
NR: Nicotinamide riboside
NMN: Nicotinamide mononucleotide
NAM: Nicotinamide
SARM1: Sterile alpha and TIR motif containing 1
ARTC1: Arginine-specific ecto-ADP-ribosyltransferase
FASEB: Federation of American Societies for Experimental Biology

## References

[CR1] Berven H, Kverneng S, Sheard E. NR-SAFE: a randomized, double-blind safety trial of high dose nicotinamide riboside in Parkinson’s disease. Nat Commun. 2023;14(1):7793. 10.1038/s41467-023-43514-6. 38016950 10.1038/s41467-023-43514-6 PMC10684646

[CR2] Brakedal B, Dölle C, Riemer F, et al. The NADPARK study: A randomized phase I trial of nicotinamide riboside supplementation in parkinson’s disease. Cell Metab. 2022;34(3):396–e4076. 10.1016/j.cmet.2022.02.001. 35235774 10.1016/j.cmet.2022.02.001

[CR3] Bratkowski M, Xie T, Thayer DA, Lad S, Mathur P, Yang YS, et al. Structural and mechanistic regulation of the pro-degenerative NAD hydrolase SARM1. Cell Rep. 2020;32(5):107999. 10.1016/j.celrep.2020.107999. 32755591 10.1016/j.celrep.2020.107999

[CR4] Brenner C. Comment on Nicotinamide mononucleotide increases muscle insulin sensitivity in prediabetic women. Science. 2021;373(6554):eabj1696. 10.1126/science.abj1696. PMID: 34326206. 10.1126/science.abj1696 34326206

[CR5] Candal-Pedreira C, Rey-Brandariz J, Martín-Gisbert L, Teijeiro A, García G, Ruano-Ravina A, Pérez-Ríos M. Blue Zones, an Analysis of Existing Evidence through a Scoping Review. Aging Dis. 2025 Jun 4. https:10.14336/AD.2025.0461. Epub ahead of print. PMID: 40479568. 10.14336/AD.2025.0461 PMC13061570 40479568

[CR6] Chellappa K, McReynolds MR, Lu W, Zeng X, Makarov M, Hayat F, Mukherjee S, Bhat YR, Lingala SR, Shima RT, Descamps HC, Cox T, Ji L, Jankowski C, Chu Q, Davidson SM, Thaiss CA, Migaud ME, Rabinowitz JD, Baur JA. NAD precursors cycle between host tissues and the gut Microbiome. Cell Metab. 2022;34(12):1947–e19595. PMID: 36476934; PMCID: PMC9825113. 36476934 10.1016/j.cmet.2022.11.004 PMC9825113

[CR7] Chini CCS, Zeidler JD, Kashyap S, Warner G, Chini EN. Evolving concepts in NAD^+^ metabolism. Cell Metab. 2021;33(6):1076–87. 10.1016/j.cmet.2021.04.003. 33930322 10.1016/j.cmet.2021.04.003 PMC8172449

[CR8] Chong MC, Silva A, James PF, Wu SSX, Howitt J. Exercise increases the release of NAMPT in extracellular vesicles and alters NAD^+^ activity in recipient cells. Aging Cell. 2022. 10.1111/ACEL.13647. 35661560 10.1111/acel.13647 PMC9282849

[CR9] Damgaard MV, Nielsen TS, Basse AL. Intravenous nicotinamide riboside elevates mouse skeletal muscle NAD^+^ without impacting respiratory capacity or insulin sensitivity. iScience. 2022;25(2):103863. 10.1016/j.isci.2022.103863. 35198907 10.1016/j.isci.2022.103863 PMC8844641

[CR10] Definition of non-inferiority trial - NCI Dictionary of Cancer Terms - NCI. Accessed October 8. 2024. https://www.cancer.gov/publications/dictionaries/cancer-terms/def/non-inferiority-trial.

[CR11] Dietary Reference Intakes | health.gov. Accessed October 8. 2024. https://health.gov/our-work/nutrition-physical-activity/dietary-guidelines/dietary-reference-intakes.

[CR12] Dollerup OL, Christensen B, Svart M, et al. A randomized placebo-controlled clinical trial of nicotinamide riboside in obese men: safety, insulin-sensitivity, and lipid-mobilizing effects. Am J Clin Nutr. 2018;108(2):343–53. 10.1093/AJCN/NQY132. 29992272 10.1093/ajcn/nqy132

[CR13] Dziadosz M, Hoefemann M, Döring A, Marjańska M, Auerbach EJ, Kreis R. Quantification of NAD ^+^ in human brain with 1H MR spectroscopy at 3 T: comparison of three localization techniques with different handling of water magnetization. Magn Reson Med. 2022;88(3):1027. 10.1002/MRM.29267. 35526238 10.1002/mrm.29267 PMC9322547

[CR14] Essuman K, Summers DW, Sasaki Y, Mao X, DiAntonio A, Milbrandt J. The SARM1 Toll/Interleukin-1 receptor domain possesses intrinsic NAD^+^ cleavage activity that promotes pathological axonal degeneration. Neuron. 2017;93(6):1334–e13435. 10.1016/j.neuron.2017.02.022. PMID: 28334607; PMCID: PMC6284238. 28334607 10.1016/j.neuron.2017.02.022 PMC6284238

[CR15] Figley MD, Gu W, Nanson JD, Shi Y, Sasaki Y, Cunnea K, Malde AK, Jia X, Luo Z, Saikot FK, Mosaiab T, Masic V, Holt S, Hartley-Tassell L, McGuinness HY, Manik MK, Bosanac T, Landsberg MJ, Kerry PS, Mobli M, Hughes RO, Milbrandt J, Kobe B, DiAntonio A, Ve T. SARM1 is a metabolic sensor activated by an increased NMN/NAD + ratio to trigger axon degeneration. Neuron. 2021;109(7):1118–e113611. 10.1016/j.neuron.2021.02.009. Epub 2021 Mar 2. PMID: 33657413; PMCID: PMC8174188. 33657413 10.1016/j.neuron.2021.02.009 PMC8174188

[CR16] Fliegert R, Gasser A, Guse AH. Regulation of calcium signalling by adenine-based second messengers. Biochem Soc Trans. 2007;35(Pt 1):109–14. 10.1042/BST0350109. 17233614 10.1042/BST0350109

[CR17] Freese R, Lysne V. Niacin – a scoping review for nordic nutrition recommendations. Food Nutr Res. 2023;67. 10.29219/fnr.v67.10299. 10.29219/fnr.v67.10299 PMC10770643 38187785

[CR18] Gerdts J, Brace EJ, Sasaki Y, DiAntonio A, Milbrandt J. SARM1 activation triggers axon degeneration locally via NAD⁺ destruction. Science. 2015;348(6233):453–7. 10.1126/science.1258366. 25908823 10.1126/science.1258366 PMC4513950

[CR19] Grozio A, Mills KF, Yoshino J, et al. Slc12a8 is a nicotinamide mononucleotide transporter. Nat Metab. 2019;1(1):47. 10.1038/S42255-018-0009-4. 31131364 10.1038/s42255-018-0009-4 PMC6530925

[CR20] Hamlette D. (2024). Vitamins, Minerals & Supplements – US – 2024, http://academic.mintel.com.

[CR21] Jiang Y, Liu T, Lee CH, Chang Q, Yang J, Zhang Z. The NAD^+^-mediated self-inhibition mechanism of pro-neurodegenerative SARM1. Nature. 2020;588(7839):658–63. 10.1038/s41586-020-2862-z. Epub 2020 Oct 14. PMID: 33053563. 33053563 10.1038/s41586-020-2862-z

[CR22] Kitzmiller C. (2024). Passive Beauty – US – 2024, http://academic.mintel.com.

[CR23] Klein S, Yoshino M. Response to comment on nicotinamide mononucleotide increases muscle insulin sensitivity in prediabetic women. Science. 2021;373(6554):eabj7375. 10.1126/science.abj7375. 34326209 10.1126/science.abj7375

[CR24] Kropotov A, Kulikova V, Nerinovski K, Yakimov A, Svetlova M, Solovjeva L, et al. Equilibrative nucleoside transporters mediate the import of nicotinamide riboside and nicotinic acid riboside into human cells. Int J Mol Sci. 2021;22(3):1391. 10.3390/ijms22031391. 33573263 10.3390/ijms22031391 PMC7866510

[CR25] Lapatto HAK, Kuusela M, Heikkinen A, et al. Nicotinamide riboside improves muscle mitochondrial biogenesis, satellite cell differentiation, and gut microbiota in a twin study. Sci Adv. 2023;9(2):eadd5163. 10.1126/sciadv.add5163. 36638183 10.1126/sciadv.add5163 PMC9839336

[CR26] Leutert M, Menzel S, Braren R, Rissiek B, Hopp AK, Nowak K, et al. Proteomic characterization of the heart and skeletal muscle reveals widespread arginine ADP-ribosylation by the ARTC1 ectoenzyme. Cell Rep. 2018;24(7):1916-1929.e5. 10.1016/j.celrep.2018.07.048. 30110646 10.1016/j.celrep.2018.07.048

[CR27] Levine DC, Ramsey KM, Bass J. Circadian NAD(P)(H) cycles in cell metabolism. Semin Cell Dev Biol. 2022;126:15–26. 10.1016/j.semcdb.2021.07.008. Epub 2021 Jul 17. PMID: 34281771; PMCID: PMC8761220. 34281771 10.1016/j.semcdb.2021.07.008 PMC8761220

[CR28] Liu Y, Chen X, Deng X, et al. Association of NAD^+^ levels with metabolic disease in a community-based study. Front Endocrinol (Lausanne). 2023;14:1164788. 10.3389/FENDO.2023.1164788/FULL. 37152934 10.3389/fendo.2023.1164788 PMC10158491

[CR29] Loreto A, Di Stefano M, Gering M, Conforti L. Wallerian degeneration is executed by an NMN-SARM1-Dependent late Ca(2+) influx but only modestly influenced by mitochondria. Cell Rep. 2015;13(11):2539–52. Epub 2015 Dec 10. PMID: 26686637. 26686637 10.1016/j.celrep.2015.11.032

[CR30] Migaud ME, Ziegler M, Baur JA. Regulation of and challenges in targeting NAD^+^ metabolism. Nat Rev Mol Cell Biol. 2024;25(10):822–40. 10.1038/s41580-024-00752-w. 39026037 10.1038/s41580-024-00752-w PMC12456757

[CR31] Migliavacca E, Tay SKH, Patel HP, Sonntag T, Civiletto G, McFarlane C, et al. Mitochondrial oxidative capacity and NAD + biosynthesis are reduced in human sarcopenia across ethnicities. Nat Commun. 2019;10(1):5808. 10.1038/s41467-019-13694-1. 31862890 10.1038/s41467-019-13694-1 PMC6925228

[CR32] Nakahata Y, Sahar S, Astarita G, Kaluzova M, Sassone-Corsi P. Circadian control of the NAD + salvage pathway by CLOCK-SIRT1. Science. 2009;324(5927):654–7. 10.1126/science.1170803. Epub 2009 Mar 12. PMID: 19286518; PMCID: PMC6501775. 19286518 10.1126/science.1170803 PMC6501775

[CR33] Osterloh JM, Yang J, Rooney TM, Fox AN, Adalbert R, Powell EH, Sheehan AE, Avery MA, Hackett R, Logan MA, MacDonald JM, Ziegenfuss JS, Milde S, Hou YJ, Nathan C, Ding A, Brown RH Jr, Conforti L, Coleman M, Tessier-Lavigne M, Züchner S, Freeman MR. dSarm/Sarm1 is required for activation of an injury-induced axon death pathway. Science. 2012;337(6093):481–4. 10.1126/science.1223899. Epub 2012 Jun 7. PMID: 22678360; PMCID: PMC5225956. 22678360 10.1126/science.1223899 PMC5225956

[CR34] Payne T, Appleby M, Buckley E, et al. A double-blind, randomized, placebo-controlled trial of ursodeoxycholic acid (UDCA) in parkinson’s disease. Mov Disord. 2023;38(8):1493–502. 10.1002/mds.29450. 37246815 10.1002/mds.29450 PMC10527073

[CR35] Peek CB, Affinati AH, Ramsey KM, Kuo HY, Yu W, Sena LA, et al. Circadian clock NAD + cycle drives mitochondrial oxidative metabolism in mice. Science. 2013;342(6158):1243417. 10.1126/science.1243417. 24051248 10.1126/science.1243417 PMC3963134

[CR36] Pirinen E, Auranen M, Khan NA, et al. Niacin cures systemic NAD^+^ deficiency and improves muscle performance in Adult-Onset mitochondrial myopathy. Cell Metab. 2020;31(6):1078–e10905. 10.1016/J.CMET.2020.04.008. 32386566 10.1016/j.cmet.2020.04.008

[CR37] Postuma RB, Iranzo A, Hu M, et al. Risk and predictors of dementia and parkinsonism in idiopathic REM sleep behaviour disorder: a multicentre study. Brain. 2019;142(3):744–59. 10.1093/brain/awz030. 30789229 10.1093/brain/awz030 PMC6391615

[CR38] Presterud R, Deng WH, Wennerström AB, Burgers T, Gajera B, Mattsson K, et al. Long-term nicotinamide riboside use improves coordination and eye movements in ataxia telangiectasia. Mov Disord. 2024;39(2):360–9. 10.1002/mds.29645. 37899683 10.1002/mds.29645

[CR39] Ramsey KM, Yoshino J, Brace CS, Abrassart D, Kobayashi Y, Marcheva B, et al. Circadian clock feedback cycle through NAMPT-mediated NAD + biosynthesis. Science. 2009;324(5927):651–4. 10.1126/science.1171641. 19299583 10.1126/science.1171641 PMC2738420

[CR40] Ratajczak J, Joffraud M, Trammell SA, Ras R, Canela N, Boutant M, et al. NRK1 controls nicotinamide mononucleotide and nicotinamide riboside metabolism in mammalian cells. Nat Commun. 2016;7:13103. 10.1038/ncomms13103. 27725675 10.1038/ncomms13103 PMC5476803

[CR41] Shats I, Williams JG, Liu J, Makarov MV, Wu X, Lih FB, Deterding LJ, Lim C, Xu X, Randall TA, Lee E, Li W, Fan W, Li JL, Sokolsky M, Kabanov AV, Li L, Migaud ME, Locasale JW, Li X. Bacteria boost mammalian host NAD metabolism by engaging the deamidated biosynthesis pathway. Cell Metab. 2020;31(3):564–e5797. 10.1016/j.cmet.2020.02.001. PMID: 32130883; PMCID: PMC7194078. 32130883 10.1016/j.cmet.2020.02.001 PMC7194078

[CR42] Shi Y, Kerry PS, Nanson JD, Bosanac T, Sasaki Y, Krauss R, Saikot FK, Adams SE, Mosaiab T, Masic V, Mao X, Rose F, Vasquez E, Furrer M, Cunnea K, Brearley A, Gu W, Luo Z, Brillault L, Landsberg MJ, DiAntonio A, Kobe B, Milbrandt J, Hughes RO, Ve T. Structural basis of SARM1 activation, substrate recognition, and Inhibition by small molecules. Mol Cell. 2022;82(9):1643–e165910. 10.1016/j.molcel.2022.03.007. Epub 2022 Mar 24. PMID: 35334231; PMCID: PMC9188649. 35334231 10.1016/j.molcel.2022.03.007 PMC9188649

[CR43] Yaku K, Palikhe S, Iqbal T, Hayat F, Watanabe Y, Fujisaka S, et al. Nicotinamide riboside and nicotinamide mononucleotide facilitate NAD^+^ synthesis via enterohepatic circulation. Sci Adv. 2025;11(12):eadr1538. 10.1126/sciadv.adr1538. 40117359 10.1126/sciadv.adr1538 PMC11927621

[CR44] Yi L, Maier AB, Tao R, et al. The efficacy and safety of β-nicotinamide mononucleotide (NMN) supplementation in healthy middle-aged adults: a randomized, multicenter, double-blind, placebo-controlled, parallel-group, dose-dependent clinical trial. Geroscience. 2023;45(1):29–43. 10.1007/s11357-022-00705-1. 36482258 10.1007/s11357-022-00705-1 PMC9735188

[CR45] Yoshino M, Yoshino J, Kayser BD, et al. Nicotinamide mononucleotide increases muscle insulin sensitivity in prediabetic women. Science. 2021;372(6547):1224. 10.1126/SCIENCE.ABE9985. 33888596 10.1126/science.abe9985 PMC8550608

[CR46] Zapata-Pérez R, Wanders RJA, van Karnebeek CDM, Houtkooper RH. NAD^+^ homeostasis in human health and disease. EMBO Mol Med. 2021;13(7):e13943. 10.15252/emmm.202113943. 34041853 10.15252/emmm.202113943 PMC8261484

[CR47] Zhang J, Wang HL, Lautrup S, et al. Emerging strategies, applications and challenges of targeting NAD^+^ in the clinic. Nat Aging. 2025;5:1704–31. 10.1038/s43587-025-00947-6. 40926126 10.1038/s43587-025-00947-6

[CR48] Zhao Z, Gruszczynska-Biegala J, Zolkiewska A. ADP-ribosylation of integrin alpha7 modulates the binding of integrin alpha7beta1 to laminin. Biochem J. 2005;385(1):309–17. PMID: 15361073; PMCID: PMC1134699. 15361073 10.1042/BJ20040590 PMC1134699

[CR49] Zhao ZY, Xie XJ, Li WH, Liu J, Chen Z, Zhang B, Li T, Li SL, Lu JG, Zhang L, Zhang LH, Xu Z, Lee HC, Zhao YJ. A cell-Permeant mimetic of NMN activates SARM1 to produce Cyclic ADP-Ribose and induce Non-apoptotic cell death. iScience. 2019;15:452–66. Epub 2019 May 4. PMID: 31128467; PMCID: PMC6531917. 31128467 10.1016/j.isci.2019.05.001 PMC6531917

[CR50] Zhu XH, Lu M, Lee BY, Ugurbil K, Chen W. In vivo NAD assay reveals the intracellular NAD contents and redox state in healthy human brain and their age dependences. Proc Natl Acad Sci U S A. 2015;112(9):2876–81. 10.1073/pnas.1417921112. 25730862 10.1073/pnas.1417921112 PMC4352772

